# Characterization of Calcium Silicate Hydrate Gels with Different Calcium to Silica Ratios and Polymer Modifications

**DOI:** 10.3390/gels8020075

**Published:** 2022-01-24

**Authors:** Amirhossein Madadi, Jianqiang Wei

**Affiliations:** Department of Civil and Environmental Engineering, Francis College of Engineering, University of Massachusetts Lowell, Lowell, MA 01854, USA; Amirhossein_Madadi@uml.edu

**Keywords:** calcium silicate hydrate (CSH) gel, PAAm-co-PAA, thermal analysis, reaction rate, activation energy

## Abstract

Calcium silicate hydrate (CSH) gels, the main binding phases of hydrated cement, are the most widely utilized synthetic materials. To understand the influences of composition and polymers on the reaction kinetics and phase formation, CSH gels with varying Ca/Si ratios and amounts of poly (acrylamide-co-acrylic acid) partial sodium salt (PAAm-co-PAA) were synthesized via a direct method. The CSH gels were characterized through isothermal calorimetry, thermogravimetric analysis (TGA), X-ray diffraction (XRD), Fourier-transform infrared spectroscopy (FTIR), and Raman spectroscopy at different ages. By increasing the Ca/Si ratio from 0.8 to 1.0, the formation of CSH was enhanced with a 5.4% lower activation energy, whereas the incorporation of PAAm-co-PAA increased the temperature sensitivity of the reactions with an 83.3% higher activation energy. In the presence of PAAm-co-PAA, the reaction rate was retarded at an early age and the negative impact faded over time. The results of an XRD analysis indicated the formation of tobermorite as the main phase of the CSH gels, while the addition of PAAm-co-PAA resulted in a postponed calcium hydroxide consumption and CSH formation, which was confirmed by the decreased FTIR intensity of the C=O bond, Si–O stretching and Si–O bonds. The increased Raman vibrations of Si–O–Si bending Q^2^, Ca–O bonds, O–Si–O and asymmetric bending vibrations of SiO_4_ tetrahedra in the presence of PAAm-co-PAA indicate the intercalation of the polymeric phase and internal deformation of CSH gels.

## 1. Introduction

Portland cement, which serves as a binding phase of concrete, is the most widely employed synthetic material. Besides its excellent characteristics such as high strength and cost-effectivity, some inherent mechanical shortcomings, such as a high brittleness, low toughness, and high susceptibility toward cracking, impede a greater diversity for such material in industrial applications [1]. This remains an intractable problem that shortens the lifetime of structures and costs billions of dollars for maintenance each year [2]. According to a Vision 2020 for the concrete industry [3], USD 18–21 billion are estimated for the annual maintenance and rehabilitation of structures in the U.S. indicating a cost of USD 2.0 to USD 2.3 per cubic yard of in-place concrete. Compounding this critical issue is the fact that the manufacturing of cement also causes 8% of greenhouse gas emissions [4]. Improving the toughness and durability of cementitious materials is a route to overcome such mechanical shortcomings and to enhance the sustainability of infrastructures [5,6].

Inspired by nature, the unique strategies in toughing brittle mineral phases in living organisms pave a path towards synthesizing materials with outstanding properties, but this has not yet been fully achieved in man-made counterparts [7]. The combination of organic and inorganic ingredients with complicated hierarchical structures in biominerals and biological nanocomposites, such as the nacre of abalone shells [8,9], urchin [10,11] and limpet teeth [12] and bones [13,14], are well-made on all levels with respect to their biological function, flexibility and strength. These superior inherent characteristics result in outperforming engineering applications of such materials compared to traditional ones.

Calcium silicate hydrate (CSH) gel, the primary reaction product of cement hydration constitutes over 60% by volume of hydrated Portland cement and is responsible for the durability and strength of cement and concrete [15,16,17]. Despite its omnipresence, the structure of CSH is not yet fully elucidated. Although variable stoichiometry makes it difficult to accurately characterize the exact nature of its nanostructure [18], it has been reported that CSH gels have a three-dimensional atomic structure similar to naturally occurring tobermorite [19] with a layered geometry that mainly consists of calcium silicate sheets [20] containing chain units of silicates connected by calcium ions. Manipulating the CSH structure at a micro and nano scale by utilizing innovative methods such as bioinspired approaches has been the goal of numerous research efforts [21,22,23,24,25,26,27,28]. Among the bioinspired structural concepts, integration of CSH with organic components has been of great interest. Different studies have been performed to evaluate the possibility of modifying the structure of CSH with polymers [21,22,24,25,26,29,30,31,32]. Khoshnazar et al. [29,30] investigated the characteristics of CSH modified with nitrobenzoic acid and found that, at a low concentration of nitrobenzoic acid (0.01 mol per mol of Ca), the creep modulus and hardness of CSH systems can be improved. Matsuyama et al. [21,24,25] characterized the possibility of incorporating anionic and cationic polymers with high molecular weights into the structure of CSH at different calcium to silicate (Ca/Si) ratios. The results indicated interlayer intercalations of both types of polymers within the structure of the CSH leading to interlayer spacing expansion. The type of polymer and Ca/Si molar ratio of CSH are responsible for the intercalation characteristics. Likewise, Pelisser et al. [26,31] evaluated the addition of poly (diallyldimethylammonium chloride) during CSH synthesis using a direct precipitation method and observed intercalation of the polymer within the CSH lamellae with a decreased packing density, elastic modulus and indentation. In contrast, in an investigation of CSH modifications with neutral (PVA, PVME, PEO), cationic (PDC, PVBC) and anionic (PSS) polymers by Merlin et al. [32], no intercalation of polymeric molecules within the layered structure of the CSH at a nanoscale was observed. Instead, they reported evidence of mesoscale interactions between CSH crystallites and the polymer without any change in the CSH structural framework. Alizadeh et al. [22] also evaluated the addition of aniline during the synthesis of CSH in a two-step process and obtained a formation of CSH/polyaniline composite by polymerizing the organic monomer of aniline in the synthesized complex of the CSH/aniline. In a recent study, Starr et al. [33] synthesized and characterized a new polymer-modified CSH incorporating a styrene-butadiene rubber (SBR) binder and found a capability to control the modulus of elasticity while increasing the ultimate tensile strength capacity and toughness of the CSH gels.

In recent years, some new techniques have been utilized to synthesize the CSH/polymer nanocomposites. A layer-by-layer assembly technique was used in a study of Kamali and Ghahremaninezhad [34], who applied two types of poly(ethyleneimine) and poly(sodium 4-styrenesulfonate) (PSS) as cationic and negatively charged polymers, respectively. Through atomic force microscopy (AFM) imaging and AFM nanoindentation, a reduced packing density and an increased roughness were obtained for the CSH/polymer nanocomposites containing graphene oxide nanosheets. Through a pH-controlled self-assembly method, Picker et al. [35] synthesized CSH mesocrystals by employing PAAm-co-PAA and PVP-co-PAA copolymers as organic dispersants to form a hybrid, ordered crystalline superstructure of CSH. Their results illustrated the enhanced mechanical properties of a hybrid CSH in terms of elastic behavior, fracture toughness, and flexural strength. The selection of polymers was conducted with an objective of reducing the uncontrolled aggregation and achieving a strong interaction of the CSH/polymer [35] and two necessary main features were suggested for the polymers: (i) hydrophilic residues with amide or alcohol groups for hydrogen bond interactions, and (ii) negatively charged groups for electrostatic interactions mediated with Ca^2+^ [36]. Although a promising property improvement can be obtained from the ordered structure, the synthesis of CSH mesocrystals is still challenging due to the secondary nucleation and strong attractive forces between particles that can easily cause aggregation in a pure CSH suspension. Furthermore, the fact that a huge amount of aqueous solution is required to prepare a noticeable amount of solid CSH mesocrystals for macroscale applications indicates the necessity to improve the synthetic efficiency of the mesocrystallization method.

To the best of our knowledge, studies on the characteristics of CSH prepared with polymers with the above-mentioned features, especially at the macroscale, are very scarce. To address this critical challenge, this study aims to evaluate the effect of PAAm-co-PAA on CSH fabricated with a direct synthesis method. Four different CSH mixes with varying Ca/Si ratios and amounts of polymers were investigated. The influence of the polymer on the reaction kinetics, reactivation energy, structures, chemical and physical properties of CSH were evaluated by means of isothermal calorimetry, thermo-gravimetric analysis (TGA), powder X-ray diffraction (XRD), Fourier-transform infrared spectroscopy (FTIR), and Raman spectroscopy.

## 2. Results and Discussion

### 2.1. Hydration Kinetics

**Figure 1:**
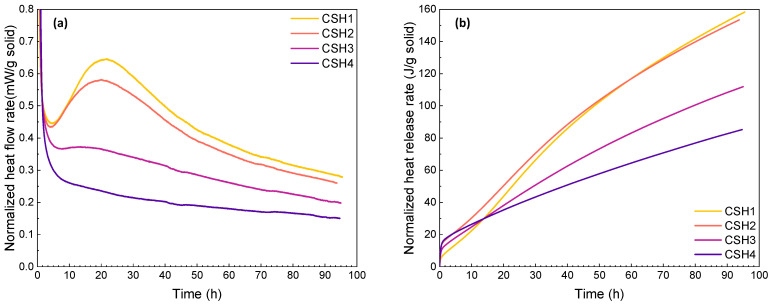
(**a**) Heat flow and (**b**) heat release rates of CSH gels measured by isothermal calorimetry at 23 °C over a reaction time of 0–96 h.

Heat flow (J/g h) and released heat (J/g) diagrams of the CSH mixtures at early ages (up to 96 h) at 23 °C are represented in Figure 1a,b, respectively. It should be noted that all the values are normalized with respect to the solid content. At 23 °C, in the initial period of a few minutes, a high heat flow rate was observed for all the mixtures. In this short period, the evolved heat might be induced by quick early-time reactions as well as non-isothermal effects in the calorimeter. Then, three hydration stages of induction, acceleration and deceleration periods occurred, where the main differences of the curves can be observed. As can be seen in Figure 1a, increasing the Ca/Si ratio from 0.8 (CSH1) to 1 (CSH2) did not have a significant effect on the induction period; however, the higher Ca/Si ratio decreased the slope of the acceleration period resulting in a lower intensity of the peak assigned to the silicate hydration. Likewise, the incorporation of the polymer (PAAm-co-PAA) decelerated the hydration process. As a result, the acceleration and deceleration period of the CSH4 disappeared with a significant decrease in the main peak intensity. This might be due to the retarded nucleation and growth of the reaction products. From Figure 1b, it can be seen that the released heat was first enhanced by increasing the Ca/Si ratio from 0.8 to 1, whereas it gradually decreased over time and became lower than the CSH with a low Ca/Si ratio after about 62 h. Similarly, the addition of PAAm-co-PAA to the CSH gel led to a higher released heat at the beginning, ending in much lower values. It can be observed that with increasing the hydration time, the difference between the final heat flow and released heat of the CSH1 and CSH2 was less than that between the CSH3 and CSH4 with PAAm-co-PAA, indicating that the change of polymer concentration had more substantial effects on the CSH hydration than the change of the Ca/Si ratio.

**Figure 2:**
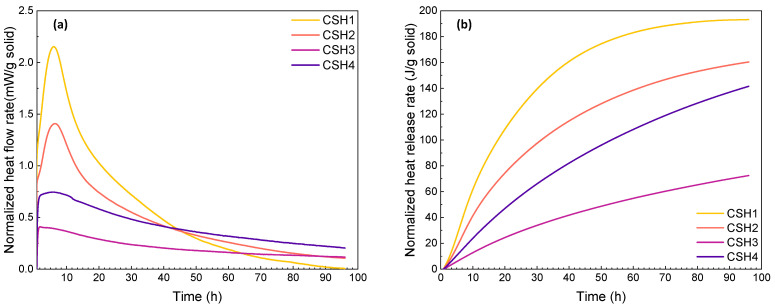
(**a**) Heat flow and (**b**) heat release rates of CSH gels measured by isothermal calorimetry at 35 °C over a reaction time of 0–96 h.

**Figure 3:**
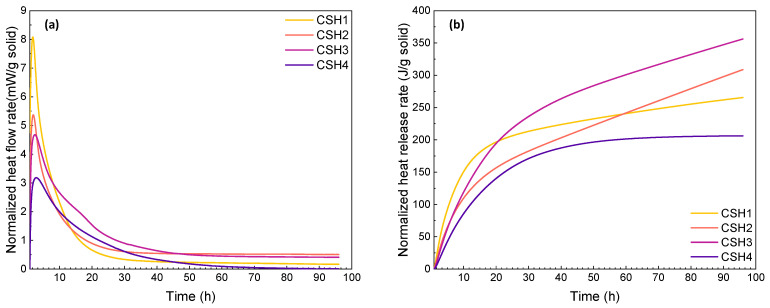
(**a**) Heat flow and (**b**) heat release rates of CSH gels measured by isothermal calorimetry at 50 °C over a reaction time of 0–96 h.

For the calorimetry tests at the two elevated temperatures, due to the difference between the chamber and ambient temperatures, a significant drop of initial heat flow was observed when placing the samples into the high-temperature chambers. To minimize the influence of the instantaneous temperature change, the hydration heat during the first 0.8 h was eliminated. Comparing the reaction heat at 23 °C, the heat flow rate as well as the peak intensity of the CSH gels increased at elevated temperatures (see Figure 2a and Figure 3a). The results indicate that an increased temperature effectively accelerates the hydration process as the main peak shifts to an earlier time. This is in agreement with the results of previous studies [37,38]. As shown in Figure 2b and Figure 3b, the enhanced reaction rates at 35 and 50 °C resulted in a raised heat release of the CSH gels with a higher increasing rate in the first 30 h. This shows that the elevated temperatures can accelerate the CSH hydration rate, which is in line with the results of other studies [39,40].

Although the heat release of the CSH1 and CSH2 at 96 h were almost the same at 23 °C, it was higher for the CSH1 at 35 °C and for the CSH2 at 50 °C, which indicates the role of the Ca/Si ratio at different temperatures. Although CSH3 as a polymer-modified CSH gel exhibited decreased heat release at 35 °C, the reaction of the CSH with a higher polymer content (CSH4) surpassed the one with less polymer (CSH3), which is opposite to the observations at 23 °C. When the temperature was further increased to 50 °C, the CSH3 yielded the highest cumulative heat lease exceeding the plain CSH gels. The comparison among the curves of heat released at different temperatures shows that, as the temperature increases, the curves of the total heat released show a higher tendency toward having a constant rate. This indicates that at elevated temperatures, due to the rapid occurrence of the reactions, the point where the CSH formation ceases and becomes ignorable for total heat released can be reached faster. This is in agreement with the results of another study [38].

### 2.2. Activation Energy

**Figure 4:**
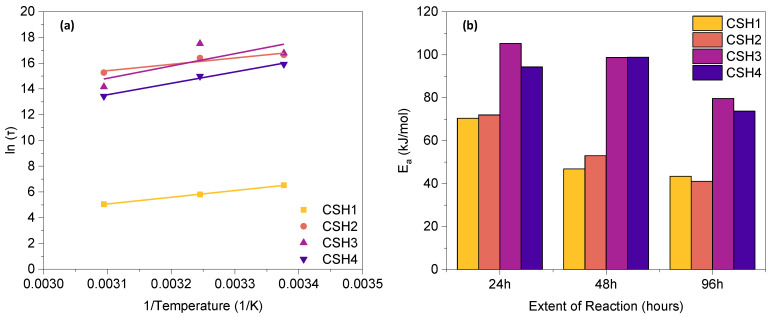
(**a**) Arrhenius plot at 96 h and (**b**) activation energy at different extent of reaction.

The Arrhenius plot for all CSH gels at 96 h and the activation energy (E_a_) at different progress points of reaction (24 h, 48 h and 96 h), are represented in Figure 4a,b, respectively. As can be seen in Figure 4b, for each CSH group, a higher E_a_ was observed at an early age, which gradually decreased with the time of reaction. The CSH3 and CSH4 exhibited a higher E_a_ than the CSH1 and CSH2, indicating that higher energy is needed for the reaction in the presence of polymer. The reason for this can be explained by the retardancy effect of the PAAm polymer which can control the speed of the hydration reaction by providing a cladding layer on the Ca(OH)_2_ to hinder the mineral contact with the water molecules [41]; however, increasing the concentration of the polymer from 8 g/L (CSH3) to 16 g/L (CSH4) resulted in a decreased E_a_.

At low reaction extents (up to 48 h), the CSH2 exhibit a higher E_a_ than the CSH1, indicating the positive role of a lower Ca/Si ratio in initiating the reactions; however, at a higher reaction extent of 96 h, the CSH2 showed a lower E_a_ than the CSH1, suggesting that less energy is needed in the presence of an elevated Ca/Si ratio and that the reaction process is better accelerated at a later age. This is in line with a previous study [38]. The higher Ca content in the CSH2 may be a reason for the decreased E_a_ due to variations of the average local energy of reactants. The magnitude of the energy barrier associated with the condensation reaction will be reduced in the presence of Ca atoms in solution, which can be the result of a competition between two effects. (i) The catalytic role of Ca atoms at the early age of gelation, which facilitates the polymerization reaction. It can be inferred that the Ca atoms can induce some nano-segregation within the gel as they like to form Ca-rich and Si-rich regions. Thus, the condensation reaction among Si(OH)_4_ monomers can be effectively facilitated as a result of such nano-segregation. Similar results were also reported in previous studies [42,43]. Moreover, the addition of Ca^2+^ cations causes an increased pH in the aqueous solution, which, in turn, results in enhanced gelation kinetics [44]. (ii) On the other hand, the high content of Ca atoms may substantially reduce the overall degree of polymerization in Ca-rich gels, leading to a hindered condensation reaction among Si(OH)_4_ monomers [45]. The dynamic competition between these two effects explains the varying E_a_ with the variations of the Ca/Si ratio at a different reaction extent (time).

By considering the entire reaction process during the first 96 h, the average E_a_ of the CSH1 and CSH2 was 43.45 and 41.11 kJ/mol, respectively, suggesting that a higher Ca/Si ratio can slightly decrease the temperature sensitivity of CSH reactions, whereas the incorporation of PAAm-co-PAA had a significant effect on increasing the temperature sensitivity so that the average E_a_ of the CSH3 and CSH4 increased to 79.65 and 73.74 kJ/mol, respectively.

### 2.3. TGA

**Figure 5:**
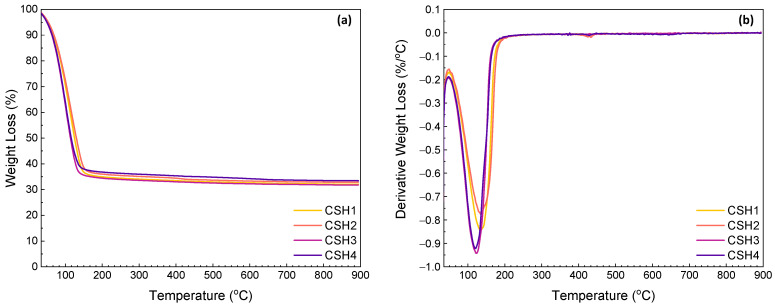
TGA (**a**) and DTG (**b**) curves for CSH gels at day 1.

**Figure 6:**
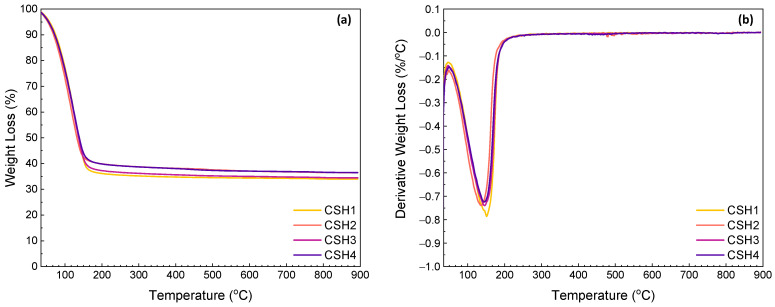
TGA (**a**) and DTG (**b**) curves for CSH gels at day 14.

Results of the TG measurements of the CSH gels after 1 and 14 days of reaction are given in Figure 5 and Figure 6, respectively. The first thermal step was observed in the range between ambient temperature and 240 °C which is related to the evaporation of the adsorbed and interlayer water (30–109 °C) and dehydration of the CSH (109–240 °C) [46,47]. At an early age (day 1), a less significant weight loss was observed from the CSH2 indicating a higher reaction rate in the presence of a 0.8 Ca/Si ratio. This is in agreement with the calorimetry results (see Figure 5a). In the presence of PAAm-co-PAA, the onset temperature of the thermal decomposition shifted to a lower value. This might be due to the higher content of free water induced by the hydrophilicity of the polymer, and this explains the lower reaction rate between the calcium hydroxide and amorphous silica. Again, this agrees well with the calorimetry results as shown in Figure 5a,b. At a later age (day 14), the influence of the Ca/Si ratio can still be observed, while a lower weight loss was obtained from the polymer-modified CSH. This might be due to the consumption of free water over time. Therefore, this later-age difference indicates the facilitated formation of CSH at a Ca/Si ratio of 0.8 and the lower CSH contents in the samples with the polymer.

The second and the third weight losses that occurred in the ranges 350–500 °C and 550–893 °C are attributed to the dehydroxylation of portlandite (CH) and the decomposition of calcium carbonate due to carbonation of the CH, respectively. According to [48], three modes of calcium carbonate, which can coexist in the ultimate carbonation state of hydrated cement, can be identified from the differential TG (DTG) traces of calcium carbonate decomposition. The decomposition of well-crystallized calcite in the range of 780–990 °C could define Mode I. Mode II is explained by the decomposition of carbonates by aragonite and vaterite at a lower temperature range of 700–780 °C. Finally, the range of 550–700 °C is related to the decomposition of amorphous calcium carbonate as Mode III. It was reported that the neat PAAm usually decomposes in multiple temperature ranges from 30 to 650 °C, where the maximum weight loss (44.6%) occurs at about 540 °C [49,50], which overlaps the thermal decomposition of calcium carbonate. The slight weight losses of the TGA and the corresponding shoulders of the DTG curves in the temperature range of 720–830 °C indicate that the carbonation amount was ignorable with a slight formation of Mode I or II calcium carbonate.

Table 1 summarizes the contents of free water, CSH, CH and CO_2_ from calcium carbonate in certain temperature ranges. From the results, it can be observed that by increasing the age of specimens, the total weight loss percentage decreased from the average amount of 67.5% for the specimens at day 1 to the average amount of 64.6% for those at day 14. The main weight losses were associated with the removal of free water and the dehydration of CSH, which were 28.5–45.2% and 20.8–35.9%, respectively. From day 1–14, the amounts of free water in all the four CSH gels decreased indicating an improved degree of reaction between the CH and amorphous silica over time. On the first day of reaction, the CSH1 showed a higher water content than the CSH2, while a reversed phenomenon was observed after 14 days. By incorporating the PAAm-co-PAA, the two polymer-modified CSH gels exhibited 23.5% and 19.6% higher free water contents than the CSH1, respectively, whereas these differences decreased to 4.4% and 2.4%, respectively, after 14 days. This again reveals the decreasing influence of the polymer on the reaction over time. The CSH2 showed a CSH content of 30.4% on day 1, while this value decreased to 27.5% after 14 days. This might be due to a test error. The other three CSH gels showed increased CSH contents with time. In line with the development of the free water contents, the polymer-modified CSH gels yielded less CSH which decreased with the amount of polymer content; however, this negative impact faded with time. The weight losses corresponding to the portlandite (CH) and calcite (CaCO_3_) were in the ranges of 0.42–1.12% and 0.47–1.11%, respectively. The absence of significant weight losses above 350 °C confirms that the CH had been almost completely consumed and that carbonation was well controlled.

### 2.4. XRD Characterization

**Figure 7:**
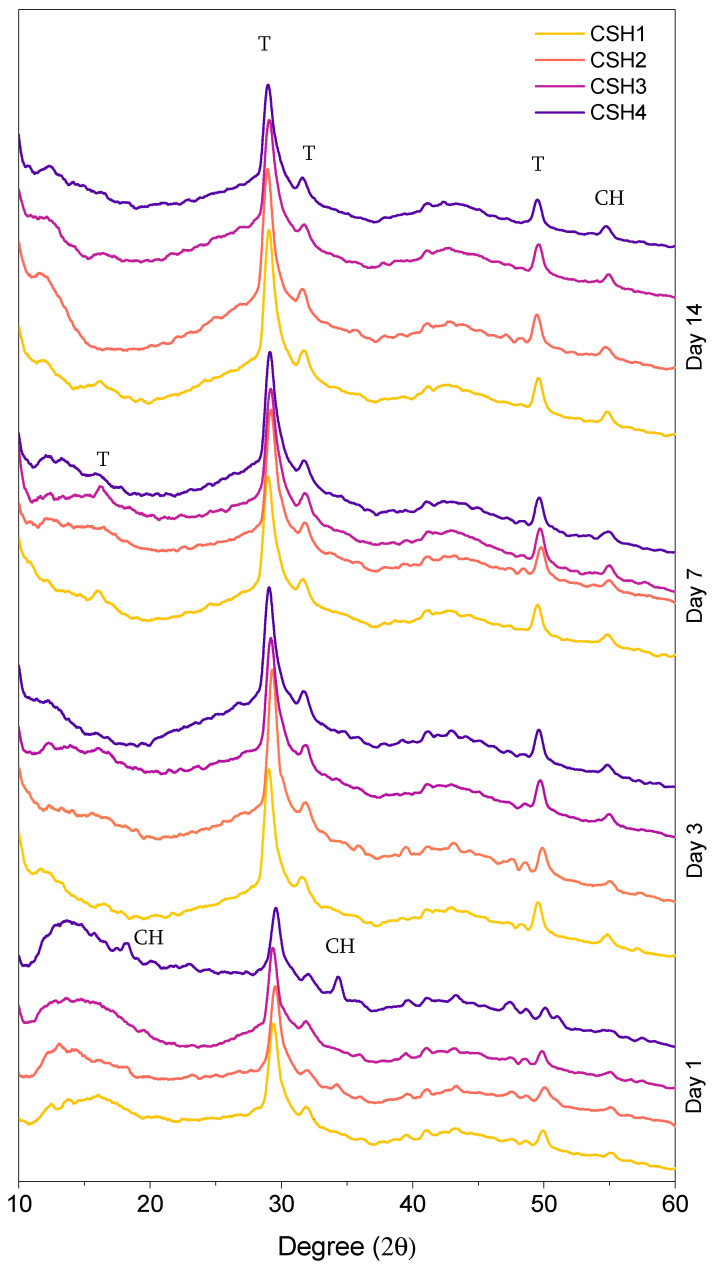
XRD pattern of different CSH gels after 1, 3, 7 and 14 days.

Figure 7 shows the wide-angle XRD pattern of the four CSH gels, which depict the formation of the crystalline structure of CH and tobermorite. It is known that the formation of tobermorite affects surface morphology [25,26]. The peaks that appeared at 29°, 32°, and 49° (2θ) were associated with the tobermorite, verifying the presence of a crystalline formation in the specimens. The CH peaks with a low intensity were also observed in all CSH gels at 54.5° (2θ), confirming the presence of a little content of unreacted CH. As can be seen, the specimens at the early age typically included the lowest intensity of tobermorite peaks, which increased with age. At each age, there was not any significant difference observed between the tobermorite peak intensity of the CSH1 and CSH2, indicating that the change of the Ca/Si ratio did not include important effects on the tobermorite formation, especially at 14 days. Comparing the pattern of the CSH1 with those of the CSH3 and CSH4, revealed a slightly higher peak intensity for the polymer modified CSH gels than the plain CSH at day 1, which gradually reversed as the age of specimens increased to 14 days. This elucidates the positive effect of the PAAm-co-PAA on early age CSH nucleation and formation which gradually fades with time.

As can be seen in Figure 7, there were some less intense features around 16.9° of the specimens of CSH1 and CSH3 at day 7. These peaks were also related to tobermorite. The specimen of CSH4 at day 1 illustrated some peaks around 18° and 34° which were associated with the CH. This shows that the addition of a higher content of PAAm-co-PAA retards the consumption of CH at early ages and postpones the formation of tobermorite.

The quantification results from the Rietveld refinement in Table 2 confirm the observations of the XRD analysis that the tobermorite made up the highest mass percentage of the solid crystalline phases in all specimens. Similar results were also reported in a previous study [51]. If not considering the proportions between the CSH and CH, then the Rietveld refinement data summarized in Table 2 are in good agreement with the TGA data (Table 1). It should be noted that CSH has a degree of long-range order with a multi-layer nature where the stacking and composition patterns are close to those found in tobermorite. The results of studies by Taylor [52,53] and later by Richardson [54,55,56] confirm this statement. As can be seen in Table 2, the average content of tobermorite at day 14 was higher than that at day 1, whereas this trend was reversed for the portlandite content. This indicates an increase in CSH formation with time, which is in line with the observations of other studies [57,58]. The highest portlandite percentage was 8.5%, which is related to the CSH4 at day 1, confirming the observations of some CH peaks on the XRD pattern of that specimen. After 14 days of reaction, the content of tobermorite in the CSH3 and CSH4 exceeded that of the CSH2 and became comparable to the CSH1, suggesting that the negative effect of PAAm-co-PAA on the formation of CSH decreased over time.

### 2.5. FTIR Analysis

**Figure 8:**
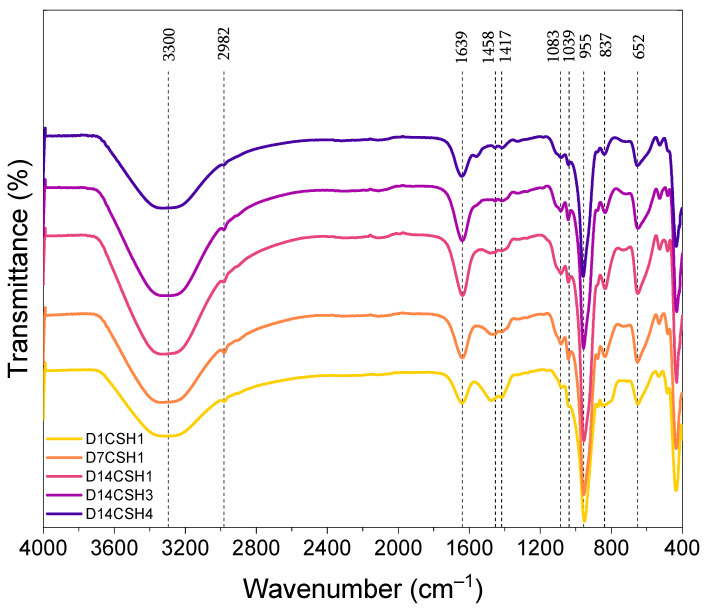
FTIR spectra of the CSH gels at different ages.

To better understand the effect of PAAm-co-PAA on CSH, the FTIR analysis was completed on the CSH1, CSH3 and CSH4, and all the spectra are depicted in Figure 8. The results represented by the bands at 1417, 1458, and 2982 cm^−1^ verify the presence of the C-H bond of polymers (PAAm-co-PAA) in the corresponding bonds. A broader band at 3300 cm^−1^ attributed to the O–H stretching frequencies was also observed. The intensity of the C=O bond at 1639 cm^−1^ increased with time, indicating an increased CSH content in the CSH1; however, at the same age (14 days), it was observed that the addition of polymer resulted in a decreased peak intensity. Agreeing well with the TGA and XRD results, this indicates that the addition of the polymer may have a negative effect on the formation of CSH. The peaks at 652, 837, and 955 cm^−1^, which were associated with the Si–O stretching band in the CSH [59], exhibited an increasing peak intensity from day 1 to those at day 14, indicating the formation of additional CSH gels over time. The addition of the polymer and increasing its content, gradually reduced the peak intensity at these bonds indicating a lower amount of CSH phases in the mixtures with polymer than for those without the polymer.

The peaks at 1039 and 1083 cm^−1^ were also observed for all spectra which were related to the asymmetric and symmetric stretching vibrations of Si–O bonds [59]. The intensity of the peaks at this bond increased from day 1 towards day 14 for the CSH1, revealing CSH formation over time. The addition of the polymer gradually decreased the peak intensity, which again confirms the retarded formation of CSH.

### 2.6. Raman Analysis

**Figure 9:**
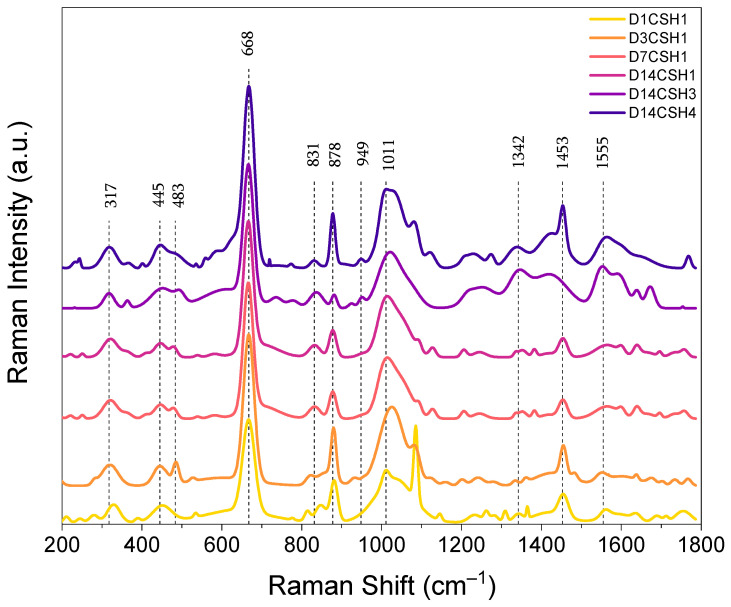
Raman spectra of the CSH gels at different ages.

The Raman bands characteristics of the CSH1 at 1 day, 3 days, 7 days and 14 days, and that of the CSH3 and CSH4 at 14 days, in the frequency range of 200–1800 cm^−1^, are illustrated in Figure 9. The highest peak intensity in the spectra of all specimens was observed at the peak located at about 668 cm^−1^, which can be attributed to the vibrations of Si–O–Si_symmetric_ bending (SB) Q^2^ [26]. The increasing peak intensity over time indicates the formation of additional CSH gel in the sample of the CSH1. The CSH4, which contained 16 g/L of PAAm-co-PAA, showed the highest peak intensity among all the CSH gels, which might be related to a lower degree of silicate chain polymerization at this mix [46,60,61]. The wavenumbers in the range of 150–400 cm^−1^ were related to the vibrational modes of calcium [62]. The main peak in this region at around 317 cm^−1^ can be observed in the spectra of the three CSH gels as evidence of Ca–O bonds. In line with the Si–O–Si bending, the peak intensity of Ca–O bonds increased with time in the CSH1 and also increased with the incorporation of the PAAm-co-PAA.

Two peaks at about 445 cm^−1^ and 483 cm^−1^ associated with υ2-type internal deformations (O_non_–Si–O_nonbending_ vibrations) and υ4-type asymmetric bending (ASB) vibrations of SiO4 tetrahedra, respectively [60,63] were observed from both the plain and polymer-modified CSH gels. As can be seen, the prominent peak at 445 cm^−1^ exhibited a decreased intensity in the CSH1 with time, while the intensity of this peak was increased in the presence of the PAAm-co-PAA (CSH3 and CSH4). In a study by Garbevet et al. [60], it was stated that the Si–O_non_–X structure can be influenced by the nearest neighbor atoms and that the change in the Ca/Si ratio can change the values of full width at half maximum (FWHM) of the peaks related to these structures. By increasing the Ca/Si ratio, Ca becomes the main constituent of the nearest neighbor atom, while in a low Ca/Si ratio, it is mainly consisting of hydrogen. The higher electronegativity of hydrogen was thus the reason for a broader, less resolved peak at a low Ca content. This is in line with literature about CSH [64].

In addition to the peaks of bonds in the CSH, inelastic scattering appeared as the bands at 831 cm^−1^ with a satellite peak of 878 cm^−1^ which were assigned to υ1(SiO_4_) vibrations [65]. A prominent peak at about 1011 cm^−1^, which was associated with υ3(SiO_4_) symmetric stretching of Q^2^ and that increased with time and polymer, was also detected. In the range of 1100 and 1200 cm^−1^, there were some minor peaks with weak bands that were mainly related to the sulfates υ2(SiO_4_) [66,67]. The band at 1011 cm^−1^ with increased intensity over time was attributed to the symmetric stretching of the sulfate ion related to the υ1(SO_4_) band [65]. There were also sharp peaks observed at 1453 cm^−1^ related to carbonate absorption bands. The main reason for the formation of these bands might be the carbonation [46,60,61,68] of the CSH gels, although it was negligible based on the TGA analysis. The peaks at 949, 1342, and 1555 cm^−1^, which were observed with higher intensities from the CSH3 and CSH4 than the CSH1, indicate the presence of PAAm-co-PAA inside the CSH gels.

## 3. Conclusions

In this study, the reaction rate and formation of CSH gels at different Ca/Si ratios and the incorporation of a polymer were investigated by isothermal calorimetry, TGA, XRD, FTIR and Raman spectrometry. The main conclusions of this study can be drawn as follows:

- The calorimetry results showed a decreased early-age hydration of the silicate phases by increasing the Ca/Si ratio and the PAAm-co-PAA content. The data at 96 h showed that the elevated Ca/Si ratio resulted in slightly lower activation energy of the reactions, while the addition of PAAm-co-PAA significantly increased the temperature sensitivity of the CSH reaction with higher activation energy.
- The decreased free water content and increased content of CSH in the gels indicate the improved degree of reaction between CH and amorphous silica over time, while the retarding influence of a 1.0 Ca/Si ratio and PAAm-co-PAA on the formation of CSH faded with time.
- The XRD analysis and Rietveld refinement indicated the formation of tobermorite as the main phase of CSH, which increased with time but decreased with the intercalation of the polymeric phase at an early age (1 day). After 14 days, the negative influence of PAAm-co-PAA on the formation of tobermorite became neglectable.
- The FTIR analysis illustrated a higher content of CSH in the specimens at higher ages. The decreased FTIR intensity of the C=O bond, Si–O stretching and Si–O bonds induced by the addition of PAAm-co-PAA indicated the negative effect of this polymer on the formation of CSH.
- The results of Raman spectroscopy confirmed the potential influence of PAAm-co-PAA on the structure modification of CSH. The increased vibrations of the Si–O–Si bending Q^2^, Ca–O bonds, O–Si–O and asymmetric bending vibrations of SiO_4_ tetrahedra in the presence of PAAm-co-PAA, indicate the intercalation of the polymeric phase and internal deformation of CSH gels.

## 4. Materials and Methods

### 4.1. Materials and Mix Designs

Extra pure calcium hydroxide (Thermo Scientific, Waltham, MA, USA, 98%), colloidal nano-silicon oxide (Thermo Scientific, 50% in water), and poly (acrylamide-co-acrylic acid) partial sodium salt (PAAm-co-PAA) (Aldrich, St. Louis, MO, USA, 80% acrylamide) with a molecular weight of 520,000 g/mol were used for the synthesis of CSH and CSH-polymer nanocomposites.

As shown in Table 3, four different mixes including two CSH gels with Ca/Si ratios of 0.8 and 1, two polymer-modified CSH gels with a Ca/Si ratio of 0.8, and two amounts of polymer were prepared. PAAm-co-PAA was mixed with deionized (DI) water to prepare solutions at the concentrations of 8 g/L and 16 g/L. A constant water to solid (W/S) ratio of 2 was used in all mixes.

### 4.2. Sample Preparation and Conditioning

The CSH gels were synthesized according to the target Ca/Si ratio and polymer contents at room temperature (23 ± 2 °C) by the direct synthesis method [47]. Two polymer stock solutions of PAAm-co-PAA with the designed concentrations of 8 g/L and 16 g/L were first synthesized under stirring at 600 rpm until clear solutions were obtained. Dry calcium hydroxide (CH) powders were manually mixed with colloidal nano-silica, DI water, and the PAAm-co-PAA solution for 30 s. The colloidal silica solution contained 50% water by mass, which was considered in the target W/S ratio. Then, the ingredients were mixed in a vacuum mixer at a speed of 500 rpm for 2 min to minimize carbonation and uniformly mix the samples. After obtaining homogenous mixtures, for each group, the samples were cast into four 25 × 25 × 25 mm cubic specimens. After casting, the specimens were sealed and conditioned at 50 °C in an oven until testing at 1, 3, 7, and 14 days. Prior to characterizations, the required amount of CSH powder was placed in an acetone bath to halt the hydration by removing residual water.

### 4.3. Characterization Methods

The isothermal calorimetry was carried out using an isothermal calorimeter I-Cal 2000 HPC (Calmetrix, Needham, MA, USA) at 23 °C, 35 °C and 50 °C to analyze the heat and activation energy (*E_a_*) of the reactions. Before mixing, the raw materials were conditioned in the calorimeter chambers at the target temperatures for 24 h to eliminate the influence of temperature on the reaction kinetics. After conditioning, the materials were uniformly mixed by hand for 1 min. Then, about 37.5 g of the samples were sealed in plastic containers and placed in the calorimeter chambers to record the heat of reactions for 96 h. The *E_a_* of each CSH matrix as the minimum kinetic energy demanded on a given reaction to occur was determined via Arrhenius’s theory, [69], see Equation (1), which has been proven an effective tool to uncover the temperature dependence of reactions of cementitious systems:

(1) $$k\left(T\right)=Ae^{-E_{a}/RT}$$

where *k*(*T*) is the rate constant (g/μg·h) at the absolute temperature *T* (K) at which reaction occurs, *A* is the preexponential factor, *E_a_* is the apparent activation energy (kJ/mol), and *R* is the universal gas constant (8.314 J/(mol·K)).

Based on a modified ASTM C1074 method [70,71], the *E_a_* was calculated as follows:

(i) The degree of hydration was modeled using Equation (2):
  (2) $$\alpha \left(t\right)=\alpha_{u}\cdot e^{-{\left[\frac{\tau }{t}\right]}^{\beta }}$$
  where *α*(*t*) is the degree of hydration at time *t*, and the three coefficients, *α_u_*, *τ*, and *β* are the ultimate degree of hydration, hydration time parameter, and hydration shape parameter, respectively.
(ii) The data of degree of hydration at each of the three temperatures were fitted by Equation (2) and the three coefficients of the model were obtained. Since *α_u_* and *β* are independent of temperature [72,73], they were obtained at 23 °C and kept constant for 35 °C and 50 °C. At each temperature, the value of *τ* was re-calculated.
(iii) The data of *ln* (*τ*) was plotted versus 1/Temperature (K) by using Equation (3):
  (3) $$E_{a}=-\frac{ln\left(\frac{\tau_{ref}}{\tau }\right)}{\frac{1}{T_{ref}}-\frac{1}{T}}\cdot R$$
  where *τ_ref_* and *τ* are hydration time parameters at reference (23 °C) and desired temperatures, respectively; *T_ref_* and *T* are the reference and desired temperatures (K), respectively.
(iv) The slope of the best-fitting line was obtained for each CSH group and multiplied by R to calculate the value of *E_a_*.

TGA was performed using a Perkin Elmer TGA4000 thermogravimetric analyzer (Perkin Elmer, Shelton, CT, USA) with an inert environment created by a 20 mL/min flow rate of nitrogen. Approximately 15 mg of each specimen was tested with a 15 min isothermal step at 30 °C, followed by a 15 °C/min heating rate from 30–900 °C.

Crystalline structures of the specimens were identified by the XRD using a Proto AXRD powder X-ray diffractometer (Proto, Taylor, MI, USA) operated with Cu Kα radiation (λ = 1.54 Å). The samples were scanned on a rotary support in stepwise mode with a step size of 0.02° (2θ) and a scanning time of 5 s per step from 10 to 60° (2θ) at an accelerated voltage of 30 kV and applied current of 20 mA.

Rietveld refinement was conducted on the specimens to identify the crystalline phases. The reported crystal structure of different available phases was used as the input phase models. The tobermorite model of [74], calcite model of [75] and portlandite model of [76] were utilized for the refinement process as they were fitting the raw XRD data better than the other available models. The background was refined using a 4th-order-polynomial, and the dominant air scattering was accounted for by refining a pseudo-Voigt peak function which was used to refine the broad diffraction peak of amorphous silica (from 15 to 30°) as well. Since tobermorite and portlandite were the two main crystalline phases detected from the XRD, the mass fractions of these two phases were refined as the core information.

The interaction between the polymer and the CSH was analyzed based on attenuated total reflection FTIR spectroscopy using a Thermo Fisher Scientific Nicolet iS10 FTIR spectrometer (Thermo Fisher, Waltham, MA, USA). The ATR-FTIR between 4000 and 400 cm^−1^ were acquired by the co-addition of 32 scans with a resolution of 4 cm^−1^.

Raman spectroscopy over the range of 200 cm^−1^ to 1800 cm^−1^ was conducted on the CSH1 after 1, 3, 7 and 14 days, and the CSH3 and CSH4 after 14 days using a MET-17 HORIBA LabRAM HR Evolution Raman Spectrometer (Horiba France SAS, Villeneuve d’Ascq, France). The measurements were performed under a 50× objective lens using a 532 nm excitation laser with a blaze grating of 600 gr/mm and a power of 2.8 mW with an acquisition time of 60 s. Two accumulations were collected for each sample. The as-received data was processed with LabSpec 6 Spectroscopy Suite by removing the background with the linear function and fitting the peaks with the Gaussian function.

## Figures and Tables

**Table 1:** Weight loss percentages due to thermal decompositions of the CSH gels.

Age	Specimen	Water Evaporation (%) (30–109 °C)	CSH (%) (109–240 °C)	CH (%) (350–500 °C)	CO_2_ (%) (550–893 °C)
1 day	CSH1	36.56	28.94	0.77	0.70
	CSH2	34.03	30.41	1.12	0.65
	CSH3	45.15	20.84	0.75	0.57
	CSH4	43.72	19.90	0.83	1.11
14 days	CSH1	28.52	35.86	0.42	0.47
	CSH2	33.18	27.53	0.82	0.83
	CSH3	29.77	33.54	0.68	0.65
	CSH4	29.20	31.57	1.02	0.66

**Table 2:** The refined mass percentage of crystalline phases.

Age	Specimen	Tobermorite (%)	Portlandite (%)
1 day	CSH1	98.2	1.8
	CSH2	95.5	4.5
	CSH3	97.3	2.7
	CSH4	91.5	8.5
14 days	CSH1	99.9	0.1
	CSH2	98.6	1.4
	CSH3	99.8	0.2
	CSH4	99.2	0.8

**Table 3:** Mix design information.

Mix No.	Ca/Si	W/S	Ca(OH)_2_ (g)	SiO_2_ (g)	PAAm-Co-PAA (75 g)
CSH1	0.8	2	25	50	-
CSH2	1	2	27.61	44.78	-
CSH3	0.8	2	25	50	8 g/L
CSH4	0.8	2	25	50	16 g/L

## Data Availability

The data presented in this study are available on request.
